# 1,7-Diethyl-4,10-diisopropyl­tetra­cene

**DOI:** 10.1107/S1600536811036415

**Published:** 2011-09-14

**Authors:** Chitoshi Kitamura, Hiroyuki Kano, Takeshi Kawase, Takashi Kobayashi, Hiroyoshi Naito

**Affiliations:** a Department of Materials Science and Chemistry, Graduate School of Engineering, University of Hyogo, 2167 Shosha, Himeji, Hyogo 671-2280, Japan; b Department of Physics and Electronics, Graduate School of Engineering, Osaka Prefecture University, 1-1 Gakuencho, Naka-ku, Sakai, Osaka 599-8531, Japan

## Abstract

The mol­ecule of the title compound, C_28_H_32_, is located on a crystallographic inversion center. The ethyl groups are essentially coplanar with the tetra­cene ring, making a torsion angle of −0.4 (4)°. The isopropyl groups adopt an asymmetric conformation with their terminal methyl groups positioned on opposite sides of the tetra­cene plane [the Me—C—C—C torsion angles are −22.5 (4) and 100.9 (3)°]. In the crystal, the mol­ecules adopt an arrangement without significant π–π inter­actions along the stacking direction (*y* axis).

## Related literature

For applications of tetra­cene derivatives, see: Anthony (2008 ▶). For crystallochromy, see: Klebe *et al.* (1989 ▶). For the synthesis, see: Kitamura *et al.* (2011 ▶). For structures of related alkyl-substituted tetra­cene derivatives, see: Kitamura, Abe *et al.* (2010 ▶); Kitamura, Tsukuda *et al.* (2010 ▶).
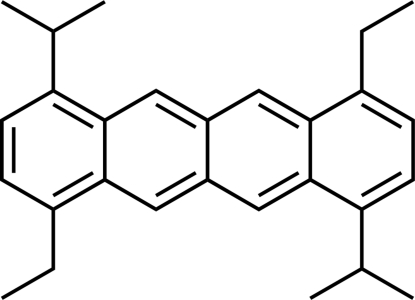


## Experimental

### 

#### Crystal data

C_28_H_32_
*M* *_r_* = 368.54
Monoclinic, 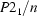

*a* = 12.901 (4) Å
*b* = 5.057 (2) Å
*c* = 16.962 (6) Å
β = 106.513 (9)°
*V* = 1061.0 (7) Å^3^
*Z* = 2
Mo *K*α radiation
μ = 0.06 mm^−1^
*T* = 203 K
0.25 × 0.13 × 0.1 mm

#### Data collection

Rigaku R-AXIS RAPID IP diffractometer
9469 measured reflections
2423 independent reflections
1318 reflections with *I* > 2σ(*I*)
*R*_int_ = 0.083

#### Refinement

*R*[*F* ^2^ > 2σ(*F* ^2^)] = 0.081
*wR*(*F* ^2^) = 0.282
*S* = 1.10
2423 reflections
130 parameters
H-atom parameters constrained
Δρ_max_ = 0.26 e Å^−3^
Δρ_min_ = −0.42 e Å^−3^

### 

Data collection: *RAPID-AUTO* (Rigaku, 1999 ▶); cell refinement: *PROCESS-AUTO* (Rigaku, 1998 ▶); data reduction: *PROCESS-AUTO*; program(s) used to solve structure: *SIR2004* (Burla *et al.*, 2005 ▶); program(s) used to refine structure: *SHELXL97* (Sheldrick, 2008 ▶); molecular graphics: *ORTEP-3 for Windows* (Farrugia, 1997 ▶); software used to prepare material for publication: *WinGX* (Farrugia, 1999 ▶).

## Supplementary Material

Crystal structure: contains datablock(s) global, I. DOI: 10.1107/S1600536811036415/ld2024sup1.cif

Structure factors: contains datablock(s) I. DOI: 10.1107/S1600536811036415/ld2024Isup2.hkl

Supplementary material file. DOI: 10.1107/S1600536811036415/ld2024Isup3.cml

Additional supplementary materials:  crystallographic information; 3D view; checkCIF report

## Figures and Tables

**Fig. 1.**
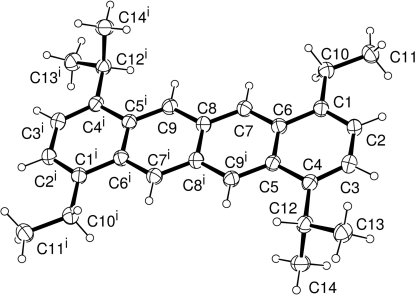
The molecular structure of the title compound, showing the atomic numbering numbering and 30% probability displacement ellipsoids for non-H atoms. Symmetry code: (i) -x, -y + 1, -z.

**Fig. 2.**
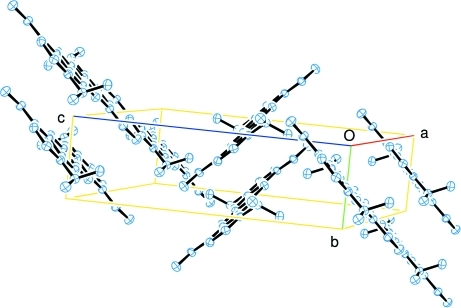
The packing diagram of the title compound. Hydrogen atoms are omitted for clarity.

## supplementary crystallographic information

### Comment

Tetracene is a promising organic semiconducting molecule for OFETs, OLEDs, and solar cells (Anthony, 2008). In addition, we have recently found that alkyl-substituted tetracenes possess interesting chromophore properties. Depending on the length, shape, and the number of alkyl side chains, the solid-state color of the tetetracenes varies through yellow, orange and red (Kitamura, Abe *et al.*, 2010; Kitamura, Tsukuda *et al.*, 2010). The difference in color can be attributed to crystallochromy (Klebe, *et al.*, 1989), *i.e.* to a color change caused by different molecular interactions based on different molecular arrangements induced by the substituents. Very recently, we have prepared *anti*/*syn*-regioisomeric mixtures of alkyl-substituted tetracenes and reported that the solid-state color of the mixtures changed before and after recrystallization from Et_2_O (Kitamura, *et al.*, 2011). To further investigate the effects of alkyl side chains on the solid-state colorations, we have synthesized an *anti*/*syn* mixture of ethyl/isopropyl-substituted tetracene (*anti* isomer – the title compound; *syn* isomer – 1,10-diethyl-4,7-diisopropyltetracene). The molecular arrangement in the crystal of the *anti* isomer is shown on Fig. 2.

### Experimental

The *anti*/*syn* ethyl/isopropyl-substituted tetracene mixture was prepared as an orange solid (329 mg) according to the method described by Kitamura *et al.* (2011), except that 2-ethyl-5-isopropyl furan was used. Recrystallization from Et_2_O afforded a yellow solid (263 mg). ^1^H-NMR: *δ* 1.51–1.56 (m, 18H), 3.28 (q, J = 7.5 Hz, 4H), 3.92–3.99 (m, 2H), 7.23–7.28 (m, 4H), 8.89 (s, 4H), 8.95 (s, 4H); ^13^C-NMR: *δ* 14.52, 23.59, 26.63, 28.65, 120.24, 122.75, 122.99, 123.27, 137.81, 142.57; EIMS: *m*/*z* (%) 368 (100); Elemental analysis for C_28_H_32_: C, 91.25; H, 8.75. Found: C, 91.17; H, 8.89. Single crystals suitable for X-ray analysis were obtained by slow evaporation from Et_2_O.

### Refinement

All the H atoms were positioned geometrically and refined using a riding model with C—H = 0.94Å and *U*_iso_(H) = 1.2*U*_eq_(C) for aromatic C—H, C—H = 0.99Å and *U*_iso_(H) = 1.2*U*_eq_(C) for CH, and C—H = 0.97Å and *U*_iso_(H) = 1.5*U*_eq_(C) for CH_3_. The positions of methyl H atoms were optimized rotationally.

### Crystal data

**Table:**

C_28_H_32_	*F*(000) = 400
*M**_r_* = 368.54	*D*_x_ = 1.154 Mg m^−^^3^
Monoclinic, *P*2_1_/*n*	Mo *K*α radiation, λ = 0.71073 Å
Hall symbol: -P 2yn	Cell parameters from 3820 reflections
*a* = 12.901 (4) Å	θ = 3.3–27.5°
*b* = 5.057 (2) Å	µ = 0.06 mm^−^^1^
*c* = 16.962 (6) Å	*T* = 203 K
β = 106.513 (9)°	Prism, yellow
*V* = 1061.0 (7) Å^3^	0.25 × 0.13 × 0.1 mm
*Z* = 2	

### Data collection

**Table:**

Rigaku R-AXIS RAPID IP diffractometer	1318 reflections with *I* > 2σ(*I*)
Radiation source: fine-focus sealed tube	*R*_int_ = 0.083
graphite	θ_max_ = 27.5°, θ_min_ = 3.3°
Detector resolution: 10 pixels mm^-1^	*h* = −16→16
ω scans	*k* = −6→6
9469 measured reflections	*l* = −22→22
2423 independent reflections	

### Refinement

**Table:**

Refinement on *F*^2^	Primary atom site location: structure-invariant direct methods
Least-squares matrix: full	Hydrogen site location: inferred from neighbouring sites
*R*[*F*^2^ > 2σ(*F*^2^)] = 0.081	H-atom parameters constrained
*wR*(*F*^2^) = 0.282	*w* = 1/[σ^2^(*F*_o_^2^) + (0.1483*P*)^2^] where *P* = (*F*_o_^2^ + 2*F*_c_^2^)/3
*S* = 1.10	(Δ/σ)_max_ < 0.001
2423 reflections	Δρ_max_ = 0.26 e Å^−^^3^
130 parameters	Δρ_min_ = −0.42 e Å^−^^3^
0 restraints	

### Special details

**Table:**

Geometry. All s.u.'s (except the s.u. in the dihedral angle between two l.s. planes) are estimated using the full covariance matrix. The cell s.u.'s are taken into account individually in the estimation of s.u.'s in distances, angles and torsion angles; correlations between s.u.'s in cell parameters are only used when they are defined by crystal symmetry. An approximate (isotropic) treatment of cell s.u.'s is used for estimating s.u.'s involving l.s. planes.
Refinement. Refinement of *F*^2^ against ALL reflections. The weighted *R*-factor *wR* and goodness of fit *S* are based on *F*^2^, conventional *R*-factors *R* are based on *F*, with *F* set to zero for negative *F*^2^. The threshold expression of *F*^2^ > 2σ(*F*^2^) is used only for calculating *R*-factors(gt) *etc*. and is not relevant to the choice of reflections for refinement. *R*-factors based on *F*^2^ are statistically about twice as large as those based on *F*, and *R*- factors based on ALL data will be even larger.

### Fractional atomic coordinates and isotropic or equivalent isotropic displacement parameters (Å^2^)

**Table:**

	*x*	*y*	*z*	*U*_iso_*/*U*_eq_	
C1	0.2359 (2)	0.9140 (5)	−0.04992 (16)	0.0445 (7)	
C2	0.3434 (2)	0.8926 (5)	−0.01162 (17)	0.0475 (7)	
H2	0.3918	1.0027	−0.0286	0.057*	
C3	0.3853 (2)	0.7094 (5)	0.05323 (17)	0.0492 (7)	
H3	0.4606	0.7005	0.0766	0.059*	
C4	0.32137 (19)	0.5464 (5)	0.08311 (16)	0.0436 (7)	
C5	0.20585 (18)	0.5603 (5)	0.04493 (15)	0.0418 (7)	
C6	0.16283 (19)	0.7463 (4)	−0.02117 (16)	0.0428 (7)	
C7	0.05225 (19)	0.7561 (5)	−0.05762 (16)	0.0457 (7)	
H7	0.025	0.8765	−0.1008	0.055*	
C8	−0.02107 (19)	0.5923 (5)	−0.03256 (16)	0.0429 (7)	
C9	−0.13335 (19)	0.6016 (5)	−0.06961 (16)	0.0446 (7)	
H9	−0.1606	0.722	−0.1128	0.054*	
C10	0.1906 (2)	1.0987 (5)	−0.12112 (17)	0.0501 (7)	
H10A	0.1395	1.2188	−0.1063	0.06*	
H10B	0.1497	0.9937	−0.1683	0.06*	
C11	0.2731 (2)	1.2644 (6)	−0.14795 (19)	0.0597 (8)	
H11A	0.322	1.1486	−0.1658	0.09*	
H11B	0.314	1.3712	−0.1021	0.09*	
H11C	0.236	1.3789	−0.193	0.09*	
C12	0.36829 (19)	0.3617 (5)	0.15477 (17)	0.0470 (7)	
H12	0.3269	0.1943	0.1437	0.056*	
C13	0.4870 (2)	0.2951 (6)	0.16758 (19)	0.0586 (8)	
H13A	0.5306	0.452	0.1855	0.088*	
H13B	0.4974	0.2324	0.1163	0.088*	
H13C	0.5088	0.1583	0.2091	0.088*	
C14	0.3535 (2)	0.4794 (6)	0.23422 (18)	0.0586 (8)	
H14A	0.2772	0.509	0.2276	0.088*	
H14B	0.392	0.6462	0.246	0.088*	
H14C	0.3819	0.3576	0.2794	0.088*	

### Atomic displacement parameters (Å^2^)

**Table:**

	*U*^11^	*U*^22^	*U*^33^	*U*^12^	*U*^13^	*U*^23^
C1	0.0426 (14)	0.0452 (14)	0.0469 (16)	−0.0039 (11)	0.0144 (11)	−0.0033 (11)
C2	0.0424 (14)	0.0539 (15)	0.0479 (16)	−0.0092 (12)	0.0153 (12)	−0.0026 (12)
C3	0.0405 (14)	0.0496 (15)	0.0564 (18)	−0.0035 (11)	0.0120 (12)	−0.0034 (12)
C4	0.0399 (13)	0.0459 (14)	0.0436 (15)	−0.0020 (11)	0.0099 (11)	−0.0050 (11)
C5	0.0383 (13)	0.0444 (14)	0.0422 (15)	−0.0013 (10)	0.0108 (10)	−0.0027 (11)
C6	0.0423 (14)	0.0432 (14)	0.0435 (16)	−0.0015 (10)	0.0134 (11)	−0.0016 (11)
C7	0.0408 (14)	0.0498 (15)	0.0461 (16)	0.0001 (11)	0.0117 (11)	0.0043 (11)
C8	0.0389 (14)	0.0438 (14)	0.0465 (16)	−0.0009 (10)	0.0129 (11)	−0.0001 (10)
C9	0.0398 (13)	0.0459 (14)	0.0454 (16)	0.0013 (11)	0.0074 (11)	0.0048 (11)
C10	0.0486 (16)	0.0493 (15)	0.0515 (18)	−0.0053 (11)	0.0125 (13)	0.0029 (12)
C11	0.0577 (18)	0.0622 (18)	0.061 (2)	−0.0058 (14)	0.0193 (15)	0.0121 (14)
C12	0.0398 (14)	0.0497 (15)	0.0491 (17)	−0.0002 (11)	0.0087 (11)	0.0013 (12)
C13	0.0436 (16)	0.0650 (18)	0.063 (2)	0.0063 (13)	0.0088 (13)	0.0059 (14)
C14	0.0576 (18)	0.0711 (19)	0.0466 (17)	0.0016 (14)	0.0140 (13)	0.0007 (14)

### Geometric parameters (Å, °)

**Table:**

C1—C2	1.359 (3)	C9—H9	0.94
C1—C6	1.451 (3)	C10—C11	1.522 (3)
C1—C10	1.507 (4)	C10—H10A	0.98
C2—C3	1.422 (4)	C10—H10B	0.98
C2—H2	0.94	C11—H11A	0.97
C3—C4	1.362 (3)	C11—H11B	0.97
C3—H3	0.94	C11—H11C	0.97
C4—C5	1.448 (3)	C12—C13	1.522 (3)
C4—C12	1.516 (3)	C12—C14	1.534 (4)
C5—C9^i^	1.394 (3)	C12—H12	0.99
C5—C6	1.448 (3)	C13—H13A	0.97
C6—C7	1.385 (3)	C13—H13B	0.97
C7—C8	1.411 (3)	C13—H13C	0.97
C7—H7	0.94	C14—H14A	0.97
C8—C9	1.406 (3)	C14—H14B	0.97
C8—C8^i^	1.430 (5)	C14—H14C	0.97
C9—C5^i^	1.394 (3)		
			
C2—C1—C6	117.7 (2)	C11—C10—H10A	108.3
C2—C1—C10	122.9 (2)	C1—C10—H10B	108.3
C6—C1—C10	119.4 (2)	C11—C10—H10B	108.3
C1—C2—C3	122.2 (2)	H10A—C10—H10B	107.4
C1—C2—H2	118.9	C10—C11—H11A	109.5
C3—C2—H2	118.9	C10—C11—H11B	109.5
C4—C3—C2	123.0 (2)	H11A—C11—H11B	109.5
C4—C3—H3	118.5	C10—C11—H11C	109.5
C2—C3—H3	118.5	H11A—C11—H11C	109.5
C3—C4—C5	117.4 (2)	H11B—C11—H11C	109.5
C3—C4—C12	121.7 (2)	C4—C12—C13	114.0 (2)
C5—C4—C12	120.9 (2)	C4—C12—C14	110.1 (2)
C9^i^—C5—C6	118.2 (2)	C13—C12—C14	109.4 (2)
C9^i^—C5—C4	122.0 (2)	C4—C12—H12	107.7
C6—C5—C4	119.8 (2)	C13—C12—H12	107.7
C7—C6—C5	119.2 (2)	C14—C12—H12	107.7
C7—C6—C1	120.9 (2)	C12—C13—H13A	109.5
C5—C6—C1	119.8 (2)	C12—C13—H13B	109.5
C6—C7—C8	122.6 (2)	H13A—C13—H13B	109.5
C6—C7—H7	118.7	C12—C13—H13C	109.5
C8—C7—H7	118.7	H13A—C13—H13C	109.5
C9—C8—C7	122.6 (2)	H13B—C13—H13C	109.5
C9—C8—C8^i^	119.0 (3)	C12—C14—H14A	109.5
C7—C8—C8^i^	118.4 (3)	C12—C14—H14B	109.5
C5^i^—C9—C8	122.7 (2)	H14A—C14—H14B	109.5
C5^i^—C9—H9	118.7	C12—C14—H14C	109.5
C8—C9—H9	118.7	H14A—C14—H14C	109.5
C1—C10—C11	115.9 (2)	H14B—C14—H14C	109.5
C1—C10—H10A	108.3		
			
C6—C1—C2—C3	1.5 (4)	C2—C1—C6—C5	−1.4 (4)
C10—C1—C2—C3	−177.3 (2)	C10—C1—C6—C5	177.4 (2)
C1—C2—C3—C4	−1.5 (4)	C5—C6—C7—C8	0.4 (4)
C2—C3—C4—C5	1.3 (4)	C1—C6—C7—C8	178.6 (2)
C2—C3—C4—C12	−177.3 (2)	C6—C7—C8—C9	−179.9 (2)
C3—C4—C5—C9^i^	178.6 (2)	C6—C7—C8—C8^i^	−0.6 (5)
C12—C4—C5—C9^i^	−2.7 (4)	C7—C8—C9—C5^i^	179.9 (2)
C3—C4—C5—C6	−1.2 (4)	C8^i^—C8—C9—C5^i^	0.7 (5)
C12—C4—C5—C6	177.4 (2)	C2—C1—C10—C11	−0.4 (4)
C9^i^—C5—C6—C7	−0.4 (4)	C6—C1—C10—C11	−179.2 (2)
C4—C5—C6—C7	179.5 (2)	C3—C4—C12—C13	−22.5 (4)
C9^i^—C5—C6—C1	−178.6 (2)	C5—C4—C12—C13	158.9 (2)
C4—C5—C6—C1	1.3 (4)	C3—C4—C12—C14	100.9 (3)
C2—C1—C6—C7	−179.6 (2)	C5—C4—C12—C14	−77.7 (3)
C10—C1—C6—C7	−0.7 (4)		

Symmetry codes: (i) −*x*, −*y*+1, −*z*.
